# Ultrasensitive protein-level detection for respiratory infectious viruses

**DOI:** 10.3389/fimmu.2024.1445771

**Published:** 2024-12-02

**Authors:** Yuki Kobayashi, Yuta Kyosei, Ryutaro Ogawa, Kyo Okita, Teruki Yoshimura, Etsuro Ito

**Affiliations:** ^1^ Department of Biology, Waseda University, Tokyo, Japan; ^2^ Department of R&D, BioPhenoMA Inc., Tokyo, Japan; ^3^ School of Pharmaceutical Sciences, Health Sciences University of Hokkaido, Hokkaido, Japan; ^4^ Graduate Institute of Medicine, Kaohsiung Medical University, Kaohsiung, Taiwan

**Keywords:** adenovirus, influenza virus, protein detection assay, respiratory syncytial virus, thio-NAD cycling, ultrasensitive ELISA

## Abstract

Influenza virus, adenovirus, and respiratory syncytial virus cause major respiratory infections. These infections have similar initial symptoms making it difficult to differentiate them based on symptoms alone. PCR is currently used as the standard diagnostic test for these infections, however, it has its limitations such as non-specific and false-negative amplifications, high cost, and the inability to distinguish between a live or dead virus. Therefore, there is a need for alternative diagnostic methods that focus on protein. Here, we introduce TN-cyclon™, which is an enzyme-linked immunosorbent assay combined with thio-nicotinamide adenine dinucleotide cycling to amplify signals, rather than the protein itself. Using this method, we were able to detect extremely low levels of viruses such as influenza A, influenza B, adenovirus, and RS virus, with LODs of 2.96 × 10^-18^ moles/assay, 2.98 × 10^-18^ moles/assay, 2.36 × 10^-18^ moles/assay, and 3.55 × 10^-18^ moles/assay, respectively. Furthermore, we successfully detected viruses diluted with extract buffer, with a significant difference to the blank at concentrations of 3 pfu/mL for influenza A, 1000 pfu/mL for influenza B, 43.8 pfu/mL for adenovirus, and 125 pfu/mL for RS virus. This shows that our low-cost and easy-to-use technique has sufficient sensitivity in diagnosing respiratory infections.

## 1 Introduction

Influenza virus, adenovirus, and respiratory syncytial (RS) virus are the major viruses causing respiratory infections. The following is the common understanding regarding the symptoms of these infectious diseases. The typical window period for influenza between exposure to the virus and the onset of symptoms is 1 – 4 days (1). The initial symptoms are predominately non-specific, including fever, chills, and headaches. Adenovirus causes adenovirus infection, one of the respiratory tract infections (2). Onset is usually 2 – 14 days after exposure to the virus. Typical symptoms are those of a mild cold or similar to influenza (3). RS virus is a common cause of respiratory hospitalization in infants, and reinfection remains common in later life (4). The symptoms are nasal congestion, runny nose, cough, and low-grade fever (5). These suggest that it is difficult to distinguish these infections based on their symptoms alone, and thus detailed nucleic acid-level or protein-level analysis must be performed.

Although PCR is believed to be a gold standard for diagnosing these infections, it amplifies nucleic acid fractions regardless of amplification errors or whether the virus is alive or dead (6), and it has several challenges such as non-specific (i.e., false-positive) and false-negative amplifications, high cost, and difficulties in designing probe sequences (7, 8). Thus, alternative diagnostic methods such as an ultrasensitive antigen test, which is more sensitive than an immunochromatographic rapid test, are required for precise diagnoses (9). To develop this, we applied an ultrasensitive enzyme-linked immunosorbent assay (ELISA) combined with thio-nicotinamide adenine dinucleotide (thio-NAD) cycling for an increase in the detection sensitivity of antigens and the detection of the viruses. This detection method is called “TN-cyclon™”, and in this system, a sandwich ELISA is used in which the secondary antibody is labeled with alkaline phosphatase (ALP), and thio-NAD cycling consists of 3α-hydroxysteroid dehydrogenase (3α-HSD) and thio-NAD and NADH as the coenzymes (10).

In the present study, we describe the application of the TN-cyclon™ to the detection of recombinant proteins and inactive viruses for the above three kinds of viruses. We found that the results obtained by TN-cyclon™ showed sufficient detection sensitivity in diagnosing infection and compensated for the shortcomings of PCR.

## 2 Materials and methods

### 2.1 Reagents and chemicals

All the antibodies used in the present study were purchased from Bio Matrix Research, Inc. (Chiba, Japan). FLA-701 and FLA-2088 were used as the capture antibody and the detection antibody, respectively, for the detection of influenza virus type A. FLB-826 and F2B-3843 were used as the capture antibody and the detection antibody, respectively, for the detection of influenza virus type B. These 4 antibodies were produced against the nucleoproteins of influenza virus type A and B. AD1-1651 and AD1-2100 were used as the capture antibody and the detection antibody, respectively, for an adenovirus assay. These antibodies were produced against the hexon proteins of adenovirus. RS-398 and RS-186 were used as the capture antibody and the detection antibody, respectively, for an RS virus assay. These antibodies were produced against the fusion proteins of RS virus. Influenza A nucleoprotein (11675-V08B), Influenza B nucleoprotein (40438-V08B), and RS virus fusion protein (40037-V08B) were purchased from Sino Biological, Inc. (Beijing, China). Adenovirus hexon protein (#30R-AA006x) was purchased from Fitzgerald Industries International (Acton, MA, USA). The ALP labeling kit for the capture antibodies was purchased from Dojindo Laboratories (Kumamoto, Japan). NADH was purchased from Sigma-Aldrich (N1161-10VL; St. Louis, MO, USA), thio-NAD was from Oriental Yeast (44104001; Tokyo, Japan), and 3α-HSD (EC. 1.1.1.50) was from Asahi Kasei Pharma (T-58; Tokyo, Japan). The substrate, 17β-methoxy-5β-androstan-3α-ol 3-phosphate (A3P), was synthesized by one of the authors (T.Y.).

### 2.2 Virus

Influenza type A (A/Puerto Rico/8/34(PR8) H1N1) virus was kindly gifted by Etsuhisa Takahashi at University of Tokushima. This strain was isolated in 1934 from a human patient in Puerto Rico and was deposited by the Centers for Disease Control and Prevention. Influenza type B (B/Washington/02/2019) (GISAID isolate ID EPI_ISL_353725) virus was isolated from a clinical specimen at National Institute of Infectious Diseases of Japan and kindly gifted. Human adenovirus 6 was purchased from ATCC (VR-6; Manassas, VA, USA). This product’s format is fluid and cell lysate from infected cultures. RS virus (RSV/S/NIID/2370/14) was kindly gifted by National Institute of Infectious Diseases of Japan (11). This virus was originally isolated from a nasal swab of 3-year old female, cultured on Hep-2 cell. Virus extract buffer was provided by BioPhenoMA Inc. (Tokyo, Japan). All viruses were inactivated by this virus extract buffer before use in the experiments.

### 2.3 Ultrasensitive ELISA with thio-NAD cycling (TN-cyclon™)

**Figure 1:**
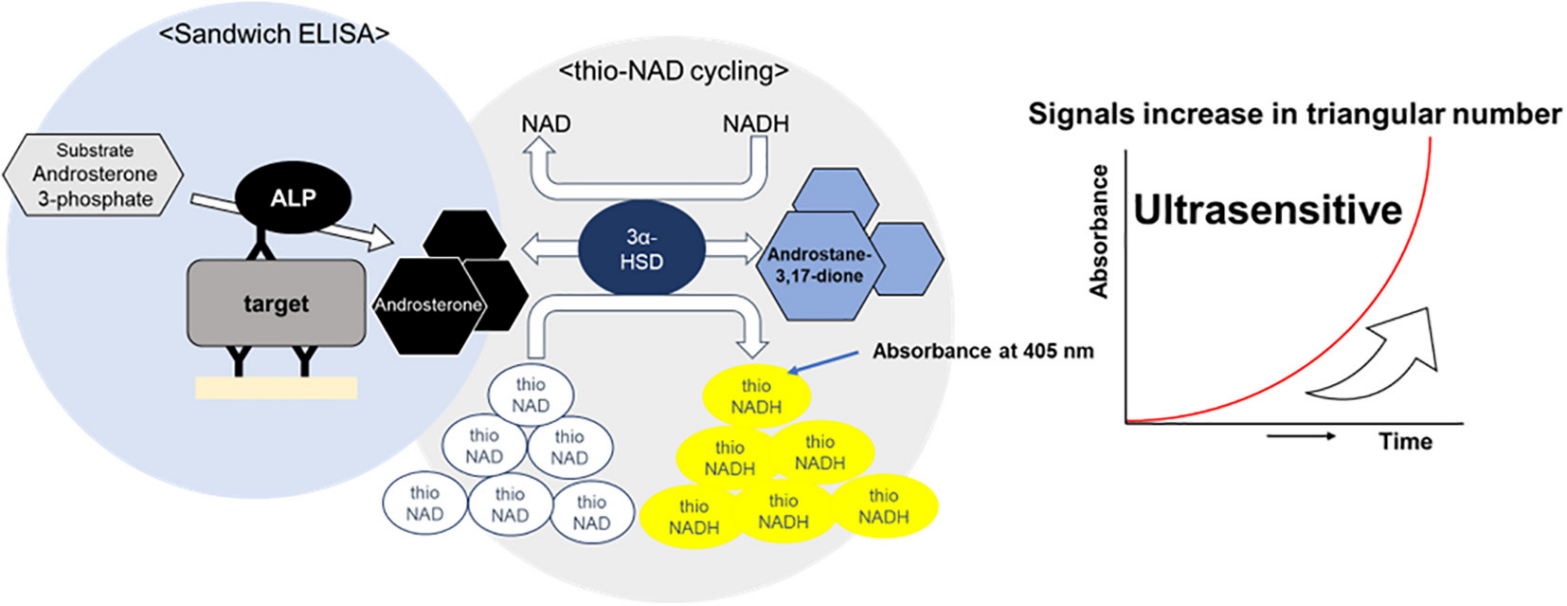
Schematic diagram of thio-NAD cycling ELISA. The signal generated by alkaline phosphatase is amplified through redox cycling via the enzyme 3α-hydroxysteroid dehydrogenase(3α-HSD) and its coenzymes, thio-NAD and NADH.

ALP labeled on the sandwich ELISA’s secondary antibody hydrolyzes 17β-methoxy-5β-androstan-3-ol 3-phosphate (Androsterone 3-phosphate) to 17β-methoxy-5β-androstan-3α-ol (i.e., Androsterone). This 17β-methoxy-5β-androstan-3α-ol is oxidized to 17β-methoxy-5β-androstan-3α-one (i.e., Androstane-3,17-dione) via 3α-hydroxysteroid dehydrogenase (3α-HSD) using thio-NAD as a coenzyme. In the reverse reaction, 17β-methoxy-5β-androstan-3α-one is reduced to 17β-methoxy-5β-androstan-3α-ol by 3α-HSD using NADH as a coenzyme. During these oxidation and reduction reactions, thio-NAD is reduced to thio-NADH during the oxidation reaction and NADH is changed to NAD during the reduction reaction. As the cycling of oxidation and reduction reactions repeats, thio-NADH accumulates in a triangular-number manner during the cycling reaction (10) (**Figure 1**). Accumulated thio-NADH can be measured directly by an increase in the absorbance at 400 nm (11,900 M^−1^ cm^−1^), e.g., 405 nm with a commercially available microplate reader, without any interference from other coenzymes such as thio-NAD, NAD and NADH, the absorbance maximums of which are all under 340 nm.

For measurements of the recombinant proteins and inactivated viruses, 100 µL of capture antibody solution diluted to 10 µg/mL in 50 mM Na_2_CO_3_ (pH 9.6) was first added to each well of microplates (469949; Thermo Scientific, Waltham, MA, USA), incubated for 1 h at room temperature, and washed 3 times with 300 µL of wash buffer consisting of 0.05% Tween20 in TBS. Next, 100 µL of 1% bovine serum albumin (BSA) in TBS was added for blocking, incubated for 1 h at room temperature, and washed 9 times with 300 µL of wash buffer. After blocking, 100 µL of recombinant protein solution, stepwise diluted in 0.1% BSA in TBS 2-fold from 1000 pg/mL to 31.25 pg/mL, was added and shaken for 1 h at room temperature. Measurements were performed in triplicates and repeated using 3 different microplates, that is, n = 9. For blanks, 100 µL of 0.1% BSA in TBS was added. It was also washed 9 times with wash buffer. The ALP-labelled detection antibody was then diluted to an antibody concentration of 100 ng/mL in TBS containing 0.1% BSA and 0.05% Tween 20, added in 100 µL portions, and shaken for 1 h at room temperature. It was also washed 9 times with wash buffer. Finally, 100 µL of 100 mM tris-HCl containing 10 U/mL 3α-HSD, 0.4 mM A3P, 1.0 mM NADH, and 2.0 mM thio-NAD were added, and measured in a microplate reader (Corona Electric SH-1000, Hitachinaka, Japan). The absorbance of 2 wavelengths was measured, 405 nm for the signal substance of thio-NADH and 660 nm for background correction, and the A_405_ – A_660_ value was used as the signal for statistical analysis. The same procedure was used to measure inactivated viruses. The viruses were diluted 2-fold from 100 pfu/mL to 3.125 pfu/mL for influenza virus A, 10^5^ pfu/mL, 10^4^ pfu/mL, 10^3^ pfu/mL, 500 pfu/mL, 250 pfu/mL, 125 pfu/mL, for influenza virus B. For adenovirus, dilutions were made 2-fold from 700 pfu/mL to 21.9 pfu/mL; for RS virus, dilutions were made 2-fold from 1000 pfu/mL to 31.25 pfu/mL. A 100 µL of each solution was added to each well. The experimental absorbance data are expressed by subtracting the blank values from the target signals at the corresponding time and the concentration data points. The calibration curves using recombinant proteins were obtained within a range of concentrations that ensured linearity of absorbance.

### 2.4 Statistical analyses

The limit of detection (LOD) was estimated by employing the mean of the blanks, the standard deviation (SD) of the blanks, and a confidence factor of 3. In the same vein, the limit of quantitation (LOQ) was approximated with the same method used for LOD, but with a confidence factor of 10. The data are expressed as the mean ± SD. The significant difference was calculated using one-way ANOVA with a *post-hoc* Holm test in R (version 4.2.2) with *P* < 0.05.

## 3 Results

### 3.1 Measurements of recombinant proteins

**Figure 2:**
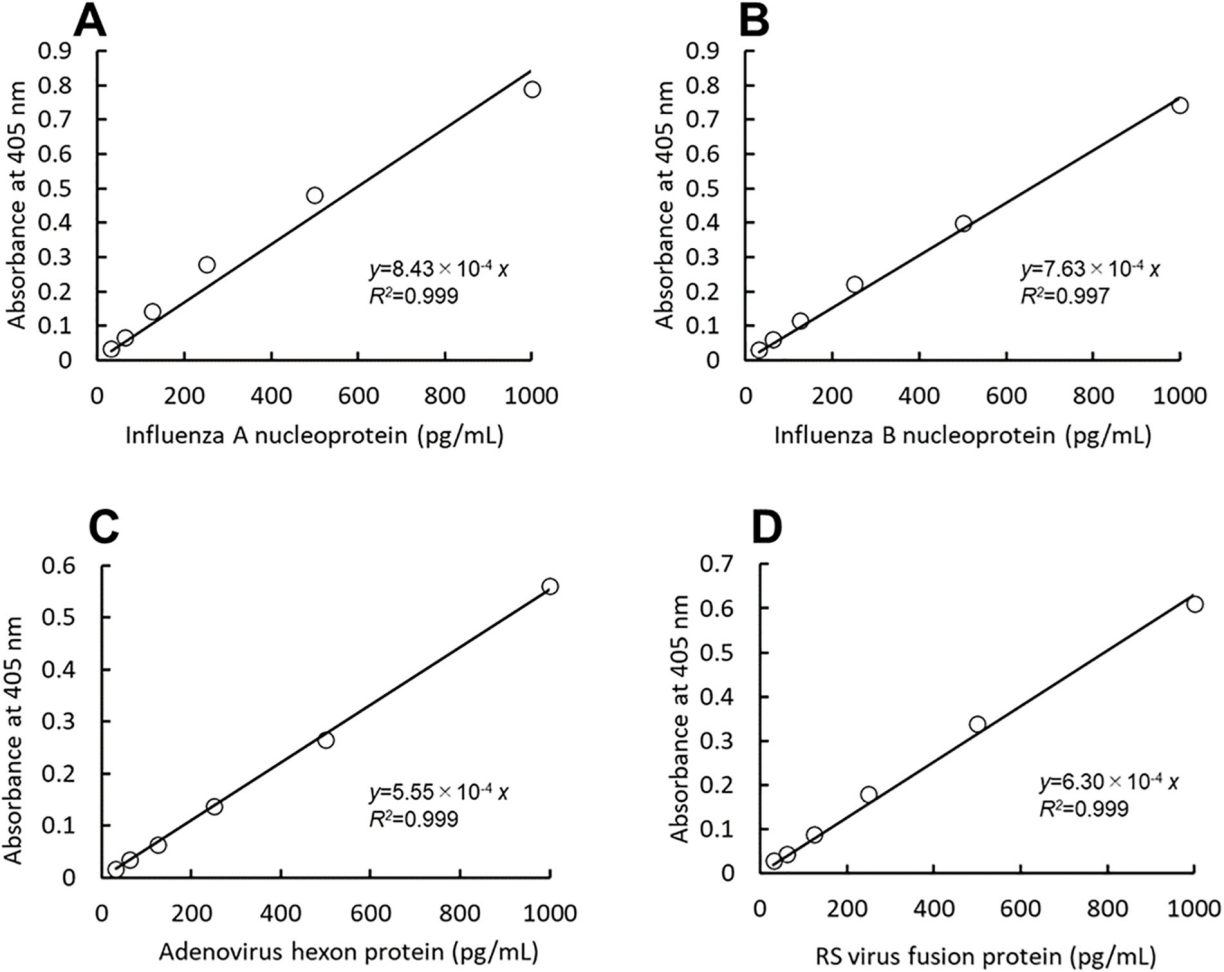
A thio-NAD cycling ELISA was used to measure the calibration curves for the nucleoproteins of influenza A **(A)** and B **(B)**, the hexon protein of adenovirus **(C)**, and the fusion protein of RS virus **(D)**. The absorbance was measured at the following time points of thio-AND cycling: 55 min for influenza A, 55 min for influenza B, 60 min for adenovirus, and 50 min for RS virus (n = 3 each). Each of the time points was determined before the saturation of the reaction absorbance. The antigen was applied within a range of 31.25 – 1000 pg/mL.

Linear calibration curves were obtained using viral recombinant proteins. The absorbance at 55 min of thio-NAD cycling was used for influenza A and B, that of 60 min was used for adenovirus, and that of 50 min was used for RS virus. The cycling time was chosen to provide optimal sensitivity. The linear calibration curves are as follows: *y* = 8.43 × 10^-4^ *x* (*R* ^2^ = 0.999) for influenza A, *y* = 7.63 × 10^-4^ *x* (*R* ^2^ = 0.997) for influenza B, *y* = 5.55 × 10^-4^ *x* (*R* ^2^ = 0,999) for adenovirus, and *y* = 6.30 × 10^-4^ *x* (*R* ^2^ = 0.999) for RS virus (**Figure 2**). The LODs were 1.68 pg/mL (2.96 × 10^-18^ moles/assay) for influenza A, 1.85 pg/mL (2.98 × 10^-18^) for influenza B, 2.55 pg/mL (2.36 × 10^-18^) for adenovirus, and 2.25 pg/mL (3.55 × 10^-18^) for RS virus. One assay volume used was 100 μL. The LOQs were 5.59 pg/mL (9.86 × 10^-18^ moles/assay) for influenza A, 6.18 pg/mL (9.95 × 10^-18^ moles/assay) for influenza B, 8.49 pg/mL (7.86 × 10^-18^ moles/assay) for adenovirus, and 7.48 pg/mL (1.18 × 10^-17^ moles/assay) for RS virus (see **Table 1** for the comparison of LOD and LOQ).

**Table 1:** LOD and LOQ of influenza type A nucleoprotein, type B nucleoprotein, adenovirus hexon protein, and RS virus fusion protein.

Virus	Antigen	LOD		LOQ	
		pg/mL	moles/assay	pg/mL	moles/assay
Influenza type A	Nucleoprotein	1.68	2.96×10^-18^	5.59	9.86×10^-18^
Influenza type B	Nucleoprotein	1.85	2.98×10^-18^	6.18	9.95×10^-18^
Adenovirus	Hexon protein	2.55	2.36×10^-18^	8.49	7.86×10^-18^
RS virus	Fusion protein	2.25	3.55×10^-18^	7.48	1.18×10^-17^

### 3.2 Measurements of viruses

**Figure 3:**
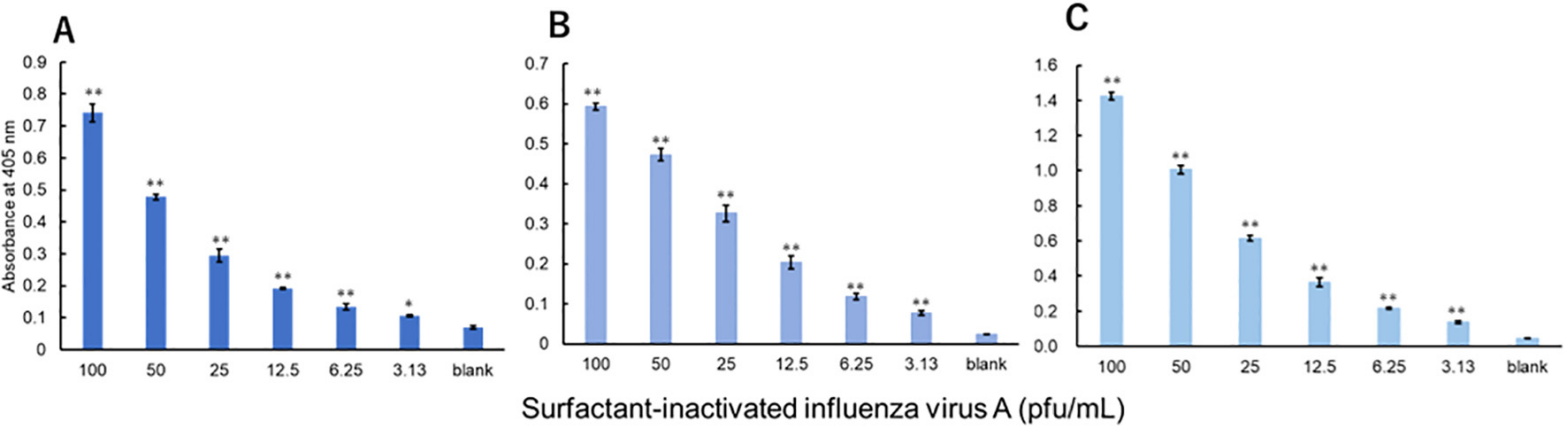
Detection of nucleoproteins in surfactant-inactivated influenza virus type A. Three datasets were measured by three investigators and are presented as **(A–C)**. Triplicate measurements were performed at 60 min of thio-NAD cycling by each investigator. All datasets show that the signals were significantly higher than the blank values at concentrations over 3.13 pfu/mL (*P < 0.05, **P < 0.01).

**Figure 4:**
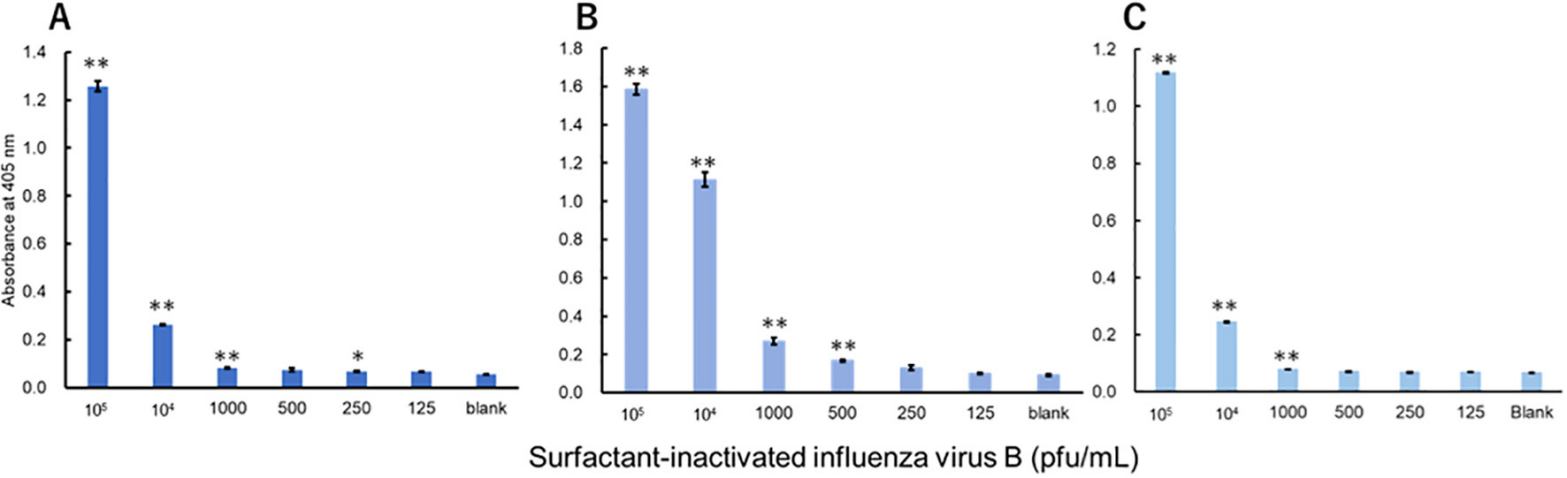
Detection of nucleoproteins in surfactant-inactivated influenza virus type B. Three datasets were measured by three investigators and are presented as **(A–C)**. Triplicate measurements were performed at 60 min of thio-NAD cycling by each investigator. All datasets show that the signals were significantly higher than the blank values at concentrations over 1000 pfu/mL (*P < 0.05, **P < 0.01).

**Figure 5:**
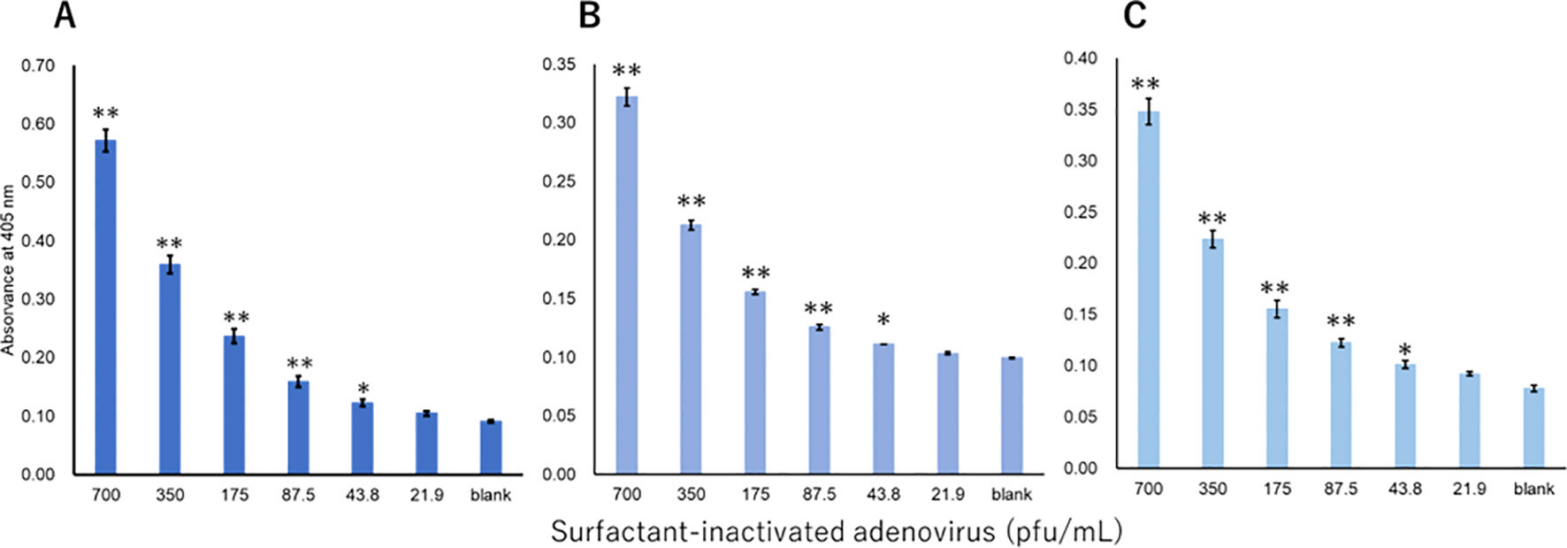
Detection of hexon proteins in surfactant-inactivated adenovirus. Three datasets were measured by three investigators and are presented as **(A–C)**. Triplicate measurements were performed at 60 min of thio-NAD cycling by each investigator. All datasets show that the signals were significantly higher than the blank values at the concentrations over 43.8 pfu/mL (*P < 0.05, **P < 0.01).

**Figure 6:**
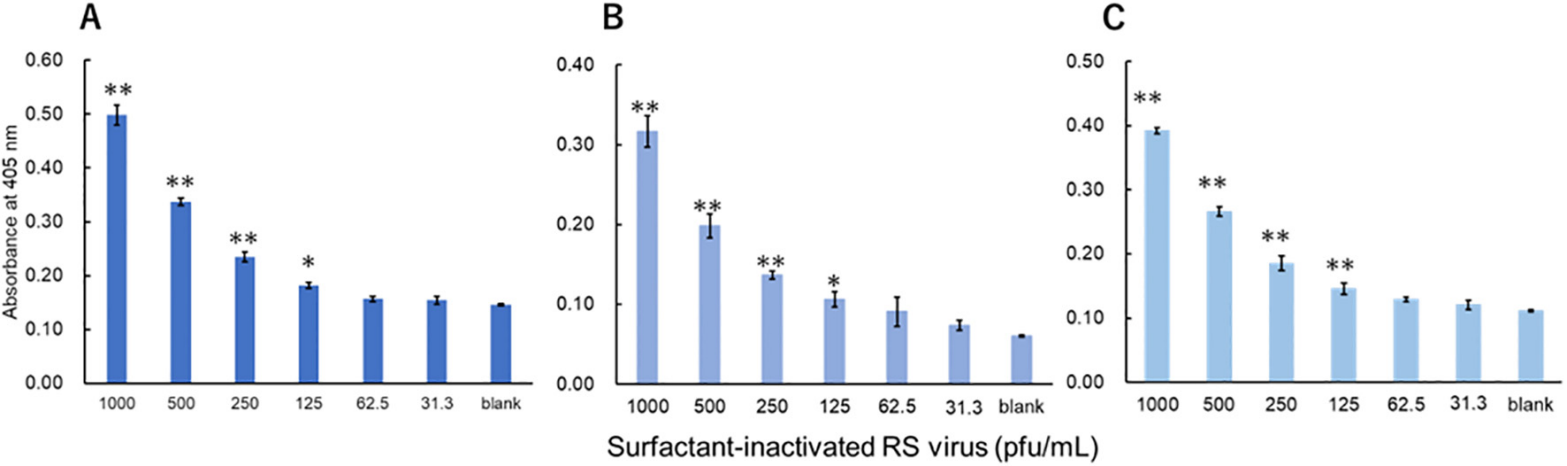
Detection of fusion proteins in surfactant-inactivated RS virus. Three datasets were measured by three investigators and are presented as **(A–C)**. Triplicate measurements were performed at 60 min of thio-NAD cycling by each investigator. All datasets show that the signals were significantly higher than the blank values at concentrations over 125 pfu/mL (*P < 0.05, **P < 0.01).

The viruses were measured using a technique equivalent to that used to measure recombinant proteins. Three investigators conducted measurements for each virus. For influenza A, all three investigators showed a significant difference in signals between 3.13 pfu/mL and the blank (**Figure 3**). For influenza B, a significant difference in signals between 1000 pfu/mL and the blank was observed by the two investigators, and that between 500 pfu/mL and the blank was observed by the one investigator (**Figure 4**). For adenovirus, all three investigators observed a significant difference in signals between 43.8 pfu/mL and the blank (**Figure 5**). For the RS virus, there was a significant difference in signals between 125 pfu/mL and the blank by all three investigators (**Figure 6**).

## 4 Discussion

### 4.1 Measurements of recombinant proteins

For the measurements of recombinant proteins, we compare our results with those of previous high-sensitivity measurements. About the measurement of recombinant influenza type A nucleoproteins, the digital ELISA assay combining ELISA and a surface plasmon resonance system had the LOD of 4 ± 1 fM [(4 ± 1) × 10^-19^ moles/100 μL] (12). An amplified luminescent proximity homogeneous assay linked immunosorbent assay (AlphaLISA) to detect influenza B nucleoproteins had an LOD of 240 pg/mL (13). It can be seen that our detection sensitivity is two orders of magnitude higher. Regarding the antibodies used in this study, the manufacturers stated that antibodies to influenza A, B, adenovirus and RS viruses do not cross-react with each other (14–17).

### 4.2 Measurements of viruses

About the previous reports for the detection of influenza A virus, combined immunomagnetic beads and biotin-nanoparticle-based detection assay targeting the nucleoproteins had the LODs of 3 × 10^3^ pfu/mL (H1N1) and 4 × 10^4^ pfu/mL (H3N2) (18). Using a fluorescence immunochromatographic assay with a quantum dot nanobead has shown that the LOD for influenza A viruses was 50 pfu/mL (19). Using a digital ELISA method has shown that the LOD for influenza A virus [A/Puerto Rico/8/1934(H1N1)] was 3.0×10^4^ pfu/mL (20). Comparing the results of these influenza A measurements, our results are comparable in sensitivity. However, our TN-cyclon™ which is modified from sandwich ELISA is overwhelmingly easy-to-use and inexpensive, so it is a very suitable measurement technology. In a study combining sandwich ELISA assay and nano-based immunosensing, adenovirus was detected with a LOD of 100 pfu/mL (21). This result is similar to ours. To our knowledge, no papers were found that discussed the detection limits for the RS virus.

### 4.3 Application of TN-cyclon™ to clarification of infection mechanism

The TN-cyclon™ method that we developed has recently begun to demonstrate its power in elucidating the infection mechanism of coronavirus disease 2019 (COVID-19). We have applied the TN-cyclon method to severe acute respiratory syndrome coronavirus 2 (SARS-CoV-2) S1 protein and achieved a LOD of 0.205 pg/mL (22). We have also applied the method to SARS-CoV-2 nucleocapsid protein and achieved a LOD of 14 pg/mL (23). Based on the results of ultrasensitive protein detection (24), women are less susceptible than men to COVID-19, which might be due to the female steroid hormone 17β-estradiol. We hypothesized that 17β-estradiol removes the soluble portion of angiotensin-converting enzyme 2 (sACE2) to which SARS-CoV-2 binds in host cells and that sACE2 then binds to the virus, thereby reducing the infectivity. Using the TN-cyclon™, we succeeded in demonstrating that the soluble portion of ACE2, which was removed from 17β-estradiol-treated VeroE6/TMPRSS2 cells, bound to the spike proteins of SARS-CoV-2, thereby reducing COVID-19 infectivity.

## 5 Conclusion

Using the TN-cyclon™ method, all recombinant proteins for infectious viruses could be measured at the pg/mL level. This is a sufficiently sensitive measurement compared to other methods. Our TN-cyclon™ method gives better or at least equal detection sensitivity to other viral detection methods.

## Data Availability

The original contributions presented in the study are included in the article/supplementary material. Further inquiries can be directed to the corresponding author.
